# A Sol–Gel Transition and Self-Healing Hydrogel Triggered via Photodimerization of Coumarin

**DOI:** 10.3390/gels10010021

**Published:** 2023-12-26

**Authors:** Yong Ye, Wenkai Wang, Xin Liu, Yong Chen, Shenghui Tian, Peng Fu

**Affiliations:** 1 School of Metallurgy and Materials Engineering, Chongqing University of Science and Technology, Chongqing 401331, China; 2 School of Materials Science and Engineering, Zhengzhou University, Zhengzhou 450001, China

**Keywords:** smart gel, sol–gel transition, self-healing, coumarin

## Abstract

Reversible chemical covalency provides a path to materials that can degrade and recombine with appropriate stimuli and which can be used for tissue regeneration and repair. However, designing and preparing efficient and quickly self-healing materials has always been a challenge. The preparation strategies of photoresponsive gels attract a lot of attention due to their precise spatial and temporal control and their predetermined response to light stimulation. In this work, the linear copolymer PAC was synthesized via precipitation polymerization of acrylic acid and 7-(2-acrylate-ethoxylated)-4-methylcoumarin. The coumarin groups on the copolymer PAC side chains provide a reversible chemical cross-linking via photostimulation, which achieves reversible regulation of the gel network structure. The concentration of 18 wt% PAC solution produces gelation under irradiation with 365 nm. In contrast, PAC gel is restored to soluble copolymers under irradiation with 254 nm. Meanwhile, the mechanical and self-healing properties of the gel were also explored. It is demonstrated that the cracks of the gel can be repaired simply, quickly, and efficiently. Furthermore, the PAC copolymer shows an excellent adhesion property based on the reversible sol–gel transition. Thus, the PAC gel has considerable potential for applications in engineering and biomedical materials.

## 1. Introduction

Smart gel refers to a kind of gel that has an obvious response to minor environmental stimuli (such as temperature, ion concentration, pressure, pH, light, electricity, magnetic field, biological ions, etc.) [1,2,3,4,5,6,7,8]. Because of its unique stimulus responsiveness, smart gel has broad application prospects in the fields of controlled release drugs, tissue engineering, artificial muscle, and sensors [9,10,11,12,13,14]. With the rapid development of biomedicine and artificial intelligence, high-intensity and smart stimulus-responsive hydrogel has attracted a lot of attention recently [15]. At present, there are two main ways to build smart gels: One is through covalent bond cross-linking. The prepared gel usually has good stability and strength and can respond to external stimuli by changing its volume and phase, but its cross-linking network is difficult to heal once destroyed [16,17,18]. The other is through intermolecular physical force. The prepared gel can realize sol–gel and self-healing under the stimulation of the external environment [19,20]. If the stability of covalently linked gel and the self-healing of physical force gel were integrated, the result would meet the requirements of more application fields such as tissue regeneration and reparation. This kind of smart gel is expected to be obtained via dynamic reversible covalent bond, which combines the advantages of covalent linked gel and physical force gel that may not only have good stability but also sol–gel transition and self-healing.

The commonly used dynamic reversible covalent bonds are imine bond, acyl bond, oxime bond, disulfide bond, Diels-Alder, etc. [21,22,23,24,25,26], while the commonly used non-covalent bonds are hydrophobic association, host and guest chemistry, hydrogen bonding, crystallization, etc. [27,28,29,30]. After external destruction, most of the current self-healing hydrogel adjusts the reversible covalent bonds of pH, temperature, etc., over a long time [20,31]. For example, Cheng Liu et al. developed a self-healing, pH-sensitive hydrogel with shape memory, taking advantage of the acylhydrazone reaction and the hydrogen bonding interaction [32]. Luke A. Connal et al. reported a doubly dynamic self-healing material based on oxime click chemistry and boronic acids, which provides a means for the synthesis of materials with tunable mechanical properties [33]. Tough and transparent polyurethane networks with self-healing capability at mild temperature conditions were successfully prepared in a one-pot procedure by Xiaoxia Jian et al. [34]. However, these stimulation methods are often difficult to achieve in the field of biomedical and tissue regeneration, which either require long-term stimulation or have negative effects on the biological system [35]. Thus, there is a need to further develop a convenient, mild, and safe self-healing hydrogel based on biocompatible matrix materials.

**Figure 1:**
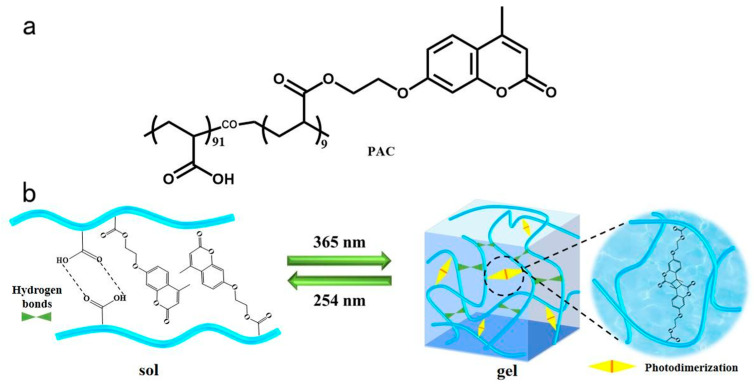
(**a**) Chemical structure of the copolymer PAC used in this study. (**b**) Schematic illustration of the reversible sol–gel transition under irradiation.

Photoresponse has great prospects among the many stimulus-response methods due to its non-physical contact, instantaneous trigger, convenient control, and simple operation [36,37,38,39]. The coumarin is known to undergo a reversible dimerization that leads to stable cyclo-butane-based dimers by irradiating (λ > 300 nm), whereas the reverse photocleavage reaction occurs under a wavelength less than 260 nm [40,41]. A series of coumarin derivatives have been designed and synthesized specifically for biofluorescent labeling [42]. Recently, light-responsive coumarin bearing amphiphilic copolymers was combined with temperature responsive micelles to obtain both mechanically strong and injectable gels by Mahinur et al. [43]. Furthermore, it is important to note that polyacrylic acid is a biocompatible material, which is an ideal polymer for constructing a biocompatible gel [44]. Herein, we develop a facile, photoresponsive, self-healing hydrogel based on copolymer poly(acrylic acid-co-7-2(acryloyl hydroxypropyl)-4-methylcoumarin) (PAC) which is a graft copolymerization of 7-2(acryloyl hydroxypropyl)-4-methylcoumarin onto acrylic acid. The copolymer PAC inherits the photoresponsiveness of coumarin and exhibits a reversible sol–gel transition via photostimulation (Figure 1). The network of PAC gel was further constructed via reversible photodimerization, physical entanglement, and hydrogen bonding, and possesses excellent mechanical properties. The self-healing properties of the hydrogel on microcracks were investigated, demonstrating its rapid self-healing properties. Moreover, based on the reversible sol–gel transition, the adhesion property of the copolymer PAC was studied as a recyclable adhesive.

## 2. Results and Discussion

### 2.1. Formation and Characterization of Coumarin-Containing Copolymer PAC

As shown in Scheme S1, firstly, 7-hydroxy-4-methylcoumarin was reacted with 2-bromoethanol in the solvent of N, N-Dimethylformamide to yield 7-(2-hydroxyethoxy)-4-methylcoumarin. The intermediate 7-(2-hydroxyethoxy)-4-methylcoumarin was further reacted with acrylic chloride to synthesize 7-(2-acrylatoethoxy)-4-methylcoumarin. Then, the linear co-polymer PAC was synthesized by polymerizing acryl acid and 7-(2-acrylatoethoxy)-4-methylcoumarin in toluene. A large amount of carboxyl and coumarin side groups of PAC helped construct the hydrogel network. Finally, the PAC hydrogel was formed via the synergistic effects of the covalent and non-covalent. The physical entanglement and hydrogen bonding between the PAC chains constructed a basic gel network. Meanwhile, the PAC chains should be further cross-linked via the photodimerization between the coumarin under UV light irradiation (λ = 365 nm). Since the covalent and non-covalent interaction of the PAC gel network are reversible, the structures and properties of PAC gel will be easier to reconstruct and regulate.

### 2.2. Photoresponsiveness of the Copolymer PAC

**Figure 2:**
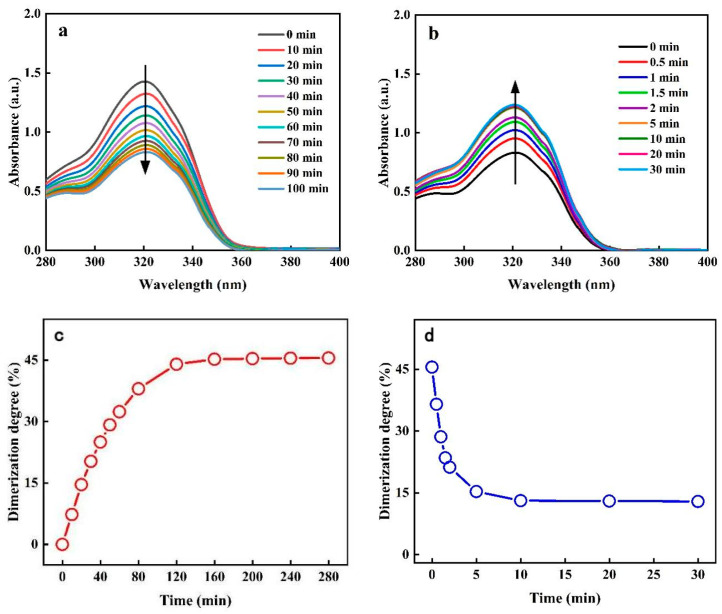
Absorption spectra of PAC solution versus time under 365 nm (**a**) and 254 nm (**b**) UV irradiation, respectively (concentration: 0.0001 g/mL); the degree of photodimerization (**c**) and photocleavage (**d**), respectively.

The photoresponse performance of PAC was first studied using a UV absorption spectrum. The time-dependent absorption spectra of PAC solutions were conducted under 365 nm UV light. As shown in Figure 2a, the characteristic absorption peak of the coumarin group still appears at 320 nm, as reported in the literature. The intensity of 320 nm gradually decreases with the length of ultraviolet irradiation due to photodimerization between the coumarin. This downward trend slowly stops after 100 min of irradiation. This demonstrates that the photodimerization between the coumarin tended towards reversible chemical equilibrium. Then, the PAC solution continued to be irradiated with 254 nm for 30 min, and the absorption intensity of peak at 320 nm was quickly restored (Figure 2b). This is attributed to the reversible photoresponse behavior of the coumarin. When the polymer PAC is irradiated with 365 nm ultraviolet light, a photocyclization addition reaction occurs between two adjacent coumarin double bonds on the polymer PAC. Since a photocleavage reaction occurs under irradiation with 254 nm ultraviolet, the cyclobutane structural bond formed by the photochemical addition reaction will break and restore the structural bond of coumarin. Moreover, this reversible photoresponse behavior of PAC exhibits a characteristic of slow photodimerization and fast photocleavage. The absorbance spectrum of PAC shows extremely different response speeds of photodimerization and photocleavage, respectively. To further investigate the pattern of photodimerization over time, the degree of photodimerization was calculated using Equation (1).

(1) $$\Delta =\frac{H_{t}-H_{0}}{\Delta T}\times 100\%$$

where H_t_ is the absorbance at the corresponding time, H_0_ is the absorbance at t = 0, and ΔT is the time interval. As shown in Figure 2c,d, the degree of dimerization gradually increased with time until reaching the maximum after 120 min, which indicates the equilibrium of photodimerization occurred. By contrast, the photocleavage quickly reached equilibrium within 10 min. Therefore, the photoresponsiveness of the PAC is characteristic of slow dimerization and fast cleavage.

**Figure 3:**
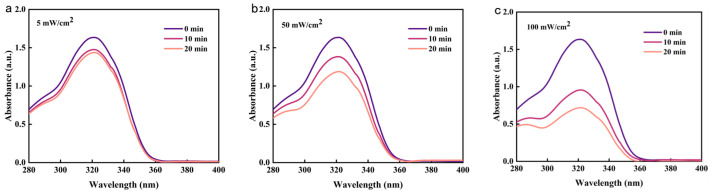

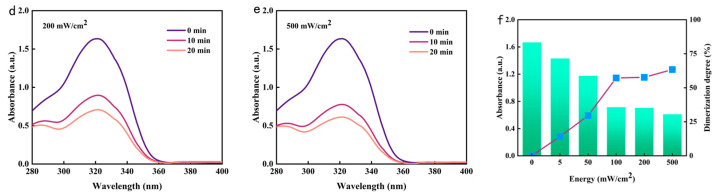
Photoresponsiveness of PAC (concentration: 0.0001 g/mL) irradiated with 365 nm UV light with an intensity of 5 mW/cm^2^ (**a**), 50 mW/cm^2^ (**b**), 100 mW/cm^2^ (**c**), 200 mW/cm^2^ (**d**), and 500 mW/cm^2^ (**e**), respectively. (**f**) Relationship between light intensity and degree of photodimerization. (The column indicates the UV absorbance, while the dotted line represents the degree of polymerization).

To investigate the stability of photoresponsiveness, the cycle of photodimerization was studied by switching the wavelength of light sources repeatedly (Figure S8). Note that the absorbance at 320 nm could not recover to the initial value and was gradually reduced after each cycle. It was speculated that a few dimers form irreversible cross-linking points. Furthermore, the intensity of ultraviolet light has a certain impact on the photodimerization efficiency and photocleavage efficiency (Figure 3). Improving the intensity can accelerate the photodimerization or photocleavage; the higher the light intensity, the significantly faster the dimerization rate. An intensity of 100 mW/cm^2^ is considered the most suitable, which can not only meet the high light dimerization efficiency but also avoid damage to the material caused by a long period of excessive light intensity.

The time-dependent emission spectra of PAC in aqueous solution were investigated to further verify the reversible photoresponse behavior (Figure S9). The concentration of PAC solution used for both fluorescent and UV spectra was 0.0001 g/mL. PAC solution exhibits weak blue fluorescence because of the coumarin. When the branched coumarins undergo a dimerization, this results in fluorescence quenched accordingly. The fluorescence intensity of PAC decreased with time under irradiation of 365 nm. While irradiated with 254 nm, the fluorescence intensity of PAC gradually increased. This is consistent with the trend of the absorption spectrum. Therefore, both absorption and emission spectra effectively demonstrated the reversible photoresponse behavior of PAC.

### 2.3. Reversible Sol–Gel Transition of PAC

**Figure 4:**
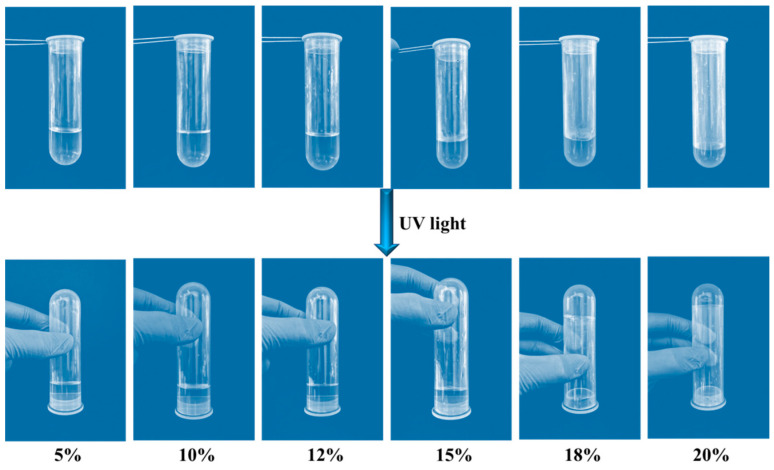
Effect of PAC concentration on the formation of sol–gel.

The influence of concentration on sol–gel transition was first studied. Due to its good hydrophilic quality, PAC exists as a free macromolecular chain in an aqueous solution. Different concentrations of PAC aqueous were prepared, and irradiated with 365 nm UV light (100 mW/cm^2^) for 20 min. As can be seen in Figure 4, when the concentration of PAC solution is below 18 wt%, the PAC solution still stays in flow after irradiation. Once above a concentration of 18 wt%, the PAC solution will change from a flow sol to a non-flow-dynamic gel. Both the non-covalent interaction and the covalent photo-cross-linking play an important role in the sol–gel process. With the concentration increasing, the non-covalent interaction is enhanced. More importantly, covalent photo-cross-linking also occurs when irradiating with 365 nm UV light. Both of these factors promoted the formation of the gel network.

**Figure 5:**
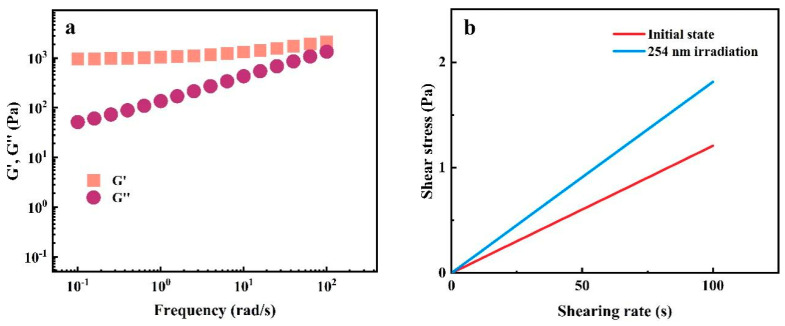
(**a**) Dynamic oscillatory frequency sweeps of 18 wt% PAC gel. (**b**) Flow curve of sol state.

To verify the state of sol and gel, the rheological test was carried out on 18 wt% PAC gel to obtain the storage modulus G′ and loss modulus G″. The storage modulus G′ is greater than the loss modulus G″, which exhibits rheological behavior typical of hydrogel (Figure 5a). Then, the PAC gel was irradiated with 254 nm UV light and its flow curve tested. Due to the small amount of polymer dispersion in the solution, the slope of its flow curve remains constant, indicating that the PAC solution at this time is a Newtonian fluid (Figure 5b). However, compared with the flow in the initial state, the slope of the flow curve is higher at this time. This is caused by the incomplete photocleavage reaction, which increases the viscosity of the sample. However, it can still be confirmed that PAC solution undergoes a reversible sol–gel transition process under light stimulation. Although PAC gel cannot be formed below the concentration of 18 wt%, the viscosity of PAC solution still rapidly rises due to a small amount of cross-linked network (Figure S10). From the above study, the minimum concentration of PAC that forms a hydrogel is 18 wt%.

**Figure 6:**
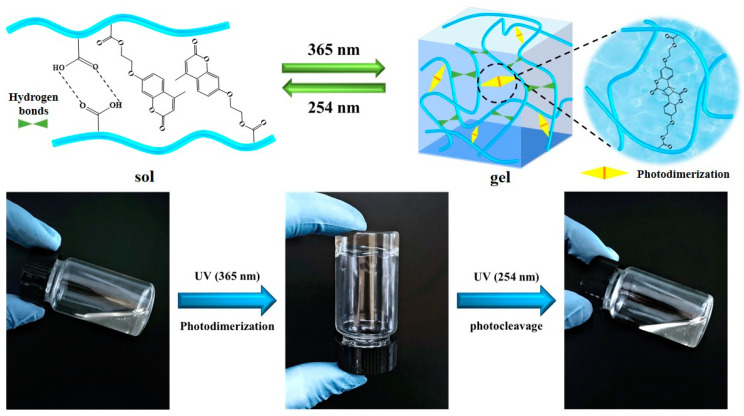
Photoresponsive reversible sol–gel transition process.

Notably, the sol–gel transition was reversible as a result of the reversible photoresponsiveness of PAC. Once irradiating the PAC gel with 254 nm ultraviolet light, the gel network will undergo cleavage of covalent cross-linking points (Figure 6). The remaining physical cross-linking points are insufficient to form a gel network, so that the gel change to sol. Then, the covalent cross-linking is formed again by irradiating sol with 365 nm ultraviolet light, and the sol will return to gel. Finally, reversible sol–gel transition was realized via photostimulation.

### 2.4. Mechanical Properties of PAC Gel

**Figure 7:**
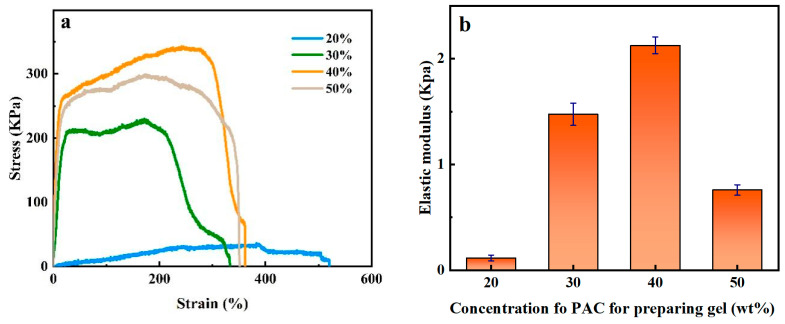
Stress–strain curves (**a**) and elastic modulus (**b**) of gels newly prepared with different PAC concentrations.

The tensile stress–strain curves of PAC gel were tested using a micro in situ mechanical testing machine. Concentrations of 20 wt%, 30 wt%, 40 wt%, and 50 wt% PAC solution were prepared as PAC gel, respectively. As shown in Figure 7a, among these samples, 20 wt% and 40 wt% PAC gel exhibited distinctive mechanical properties. The maximum tensile stress of 20 wt% PAC gel is only 33 KPa, but its tensile strain reaches 520%. In contrast, 40 wt% PAC gel shows good tensile properties with a maximum tensile stress of 340 KPa and a maximum tensile strain of 360%. However, when the concentration of PAC is higher than 40 wt%, the mechanics of the prepared gel decrease. It is speculated that excessive cross-linking points were formed in the gel, which gradually reached a “water loss” state. The gel in the “water loss” state became brittle and hard, which was accompanied by a decrease in mechanical properties. The results of the elastic modulus analysis also validate this phenomenon (Figure 7b). Thus, approximately 40 wt% PAC gel has been confirmed to possess optimal tensile properties.

Then, 40 wt% PAC gel was selected for the load–unload test. The 40 wt% PAC gel was stretched to 100%, 200%, and 300% strain respectively. The stress was then unloaded to zero at a uniform speed so that the gel slowly returned to its original state. Figure S11 shows that the dissipated energy of 40 wt% PAC gel increases with tensile strain. That energy dissipation is a result of the damage of some cross-linking networks in the gel caused by tension. The larger the tensile strain, the more networks are damaged, and the more energy is dissipated. This energy dissipation mechanism can improve the strength, toughness, fatigue resistance, and notch insensitivity of hydrogels.

### 2.5. Self-Healing Performance of the PAC Gel

**Figure 8:**
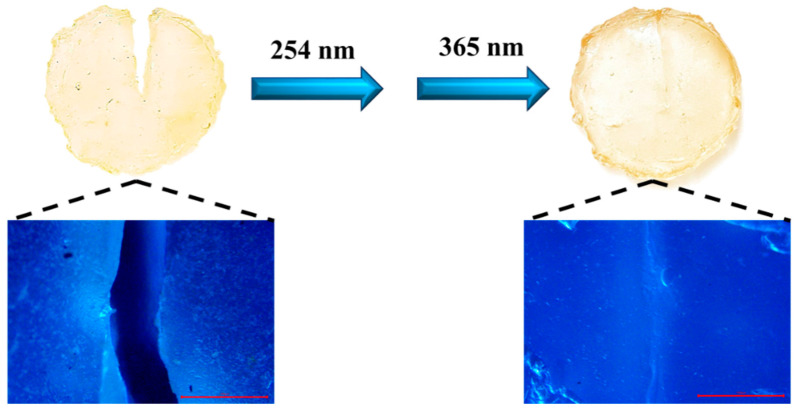
Photostimulation self-healing properties of PAC hydrogels. (The photomicrographs of repair cracks were observed under a fluorescence microscope.)

The newly prepared 40 wt% PAC gel was cut out of a crack, and we later studied its self-healing performance using photostimulation. An obvious crack was visualized under an inverted fluorescence microscope. After irradiating the wound with 365 nm UV light for a certain time, the previous crack trace was significantly reduced. This method has been further improved to produce rapid self-healing (Figure 8). The crack on the sample could be pretreated by irradiating with 254 nm UV light for 10 min, which triggered the fracture of the cross-linking points on the wound surface. Then a new cross-linking network structure was easily reconstructed in 20 min, which was irradiated with an intensity of 100 mW/cm^2^ (λ = 365 nm). In this way, the damaged wound could be rapidly healed via the reversible cross-linking reaction between the coumarin. In addition, the repair speed and efficiency increased as the thickness of the gel sample decreased. Meanwhile, loading and stretching experiments on the self-healed gel were performed to analyze the self-healing effect. The self-healing gel can carry a weight of 100 g, and its wound position was not damaged again. The tensile stress–strain curve illustrates that the self-healed gel still has good tensile properties (Figure S12). The maximum tensile stress decreases to 82% and reaches 265 KPa, and the maximum elongation at break reaches 320%. Therefore, PAC gels exhibited excellent self-healing performance, especially for rapid healing.

### 2.6. Photocontrolled Reversible Adhesion

**Figure 9:**
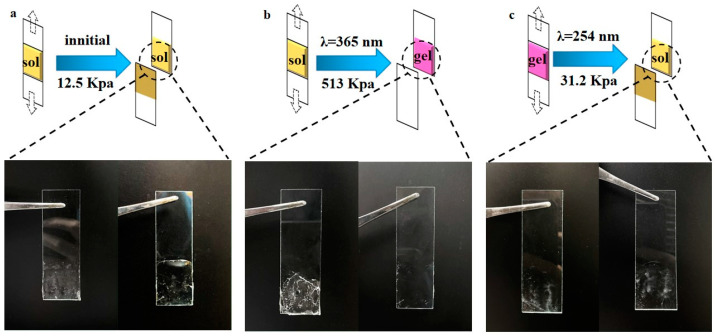
Reversible change of adhesion during sol–gel transition: (**a**) adhesion of the initial PAC sol, (**b**) adhesion of PAC gel, (**c**) adhesion of PAC sol restored from the gel.

Based on the reversible sol–gel transition of PAC, photoresponsive adhesion was explored. A 40 wt% PAC solution was prepared to assess the reversible photoresponsive adhesive strength (Figure 9). The adhesion strength of the initial state was 12.5 KPa and then increased to 513 KPa after irradiating with 365 nm UV light. The adhesion strength has increased by approximately 41 times as a result of the sol–gel transition. When using 254 nm light to stimulate gel change to the sol, the adhesive strength decreased to 31.2 KPa. The significant change in the adhesion strength is attributed to the reversible sol–gel transition. The interaction between the gel and the slide involves intermolecular interaction (Van der Waals force and hydrogen bonds) rather than the covalent bond, which can be confirmed by the phenomenon shown in Figure 9b. There was almost no gel residue on one of the slides after adhesive separation. This is due to the gel inside being cross-linked via covalent bonds and physical force when the slide and the gel were brought into contact with each other by the adhesion strength of the interface. When the gel strength was greater than the adhesion strength, the gel was almost entirely attached to one slide. However, once the gel changed to sol under the irradiation of 254 nm, the mechanical decreased significantly. The strength of the sol was less than the adhesion strength, so the sol was easily attached to the two slides. Therefore, based on the reversible photoresponsive sol–gel transition, PAC can be used as a recyclable and environmentally friendly adhesive.

## 3. Conclusions

In summary, we successfully synthesized the copolymer PAC containing coumarin, which possesses excellent adhesion and rapid self-healing properties based on reversible photoresponsive sol–gel transition. Both absorption and emission spectra effectively demonstrated the reversible photodimerization and photocleavage reaction between adjacent coumarins of PAC. Then, the sol–gel transition was shown to be reversible and efficient via illumination and rheological tests. The minimum concentration of PAC that forms a hydrogel is 18 wt%. Moreover, the prepared 40 wt% PAC gel shows excellent tensile properties and possesses a maximum tensile stress of 340 KPa and a maximum tensile strain of 360%. The PAC gel possesses a rapid self-healing behavior, completed in 20 min via photostimulation, which is firstly due to the rapid photopolymerization of coumarins, and also due to the hydrogen bonds of the carboxyl group. The maximum tensile stress of the healed 40 wt% PAC gel is 265 KPa, reaching 82% of the original. Similarly, the adhesive strength also undergoes a reversible change, increasing from an initial 12.5 KPa to 513 KPa and eventually returning to 31.2 KPa. Therefore, the photoresponsive self-healing hydrogels would be widely applied in wearable devices, adhesives, and wound dressings. Although the prepared gels can self-heal rapidly in 20 min, UV irradiation still limits their potential biomedical applications in vivo. In the future, NIR-responsive biocompatible hydrogel could be developed based on this principle.

## 4. Materials and Methods

Acrylic acid (AA, 99%, stabilized with hydroquinone methyl ether (MEHQ)), 7-Hydroxyl 4-methylcoumarin (98%), Azobisisobutyronitrile (AIBN, 98%), 2-bromoethanol (99%), potassium carbonate (K_2_CO_3_, 99%), and Acryloyl chloride (96%) were purchased from Macklin (Shanghai, China). Triethylamine (99%), dichloromethane (DCM, 99%), trichlorofluoromethane (99%), hydrochloric acid (37%), toluene (99%), and ethanol (99%) were purchased from Sinopharm Chemical Reagent Co. (Shanghai, China). All of the chemicals were used as received without further purification.

Nuclear magnetic resonance (NMR) spectra were recorded on Bruker (Billerica, MA, USA) 400 (400 MHz ^1^H) and Bruker 600 (150 MHz ^13^C) spectrometers, using CD_3_OD as solvents at room temperature. Chemical shifts were reported downfield from 0.00 ppm using TMS as the internal reference. Matrix-assisted laser-desorption ionization time-of-flight mass spectrometry (MALDI-TOF MS) was determined on the Bruker Daltonics Inc BIFLEX III MALDI-TOF (MW < 3000) mass spectrometer. The UV-Vis absorption spectra were obtained on a spectrophotometer (PE lambda 750, Fishersville, VA, USA). The corrected fluorescence spectroscopic studies were performed on a fluorescence spectrophotometer (FLS980, USA) at room temperature (25 °C). Mechanical performance tests were measured on a micro in situ mechanical testing machine (IBTC-300SL, CARE, Shenzhen, China). Rheological behavior was tested on Rheomete (MCR 102e, Leobersdorf, Austria).

### 4.1. Synthesis of 7-(2-Hydroxyethoxy)-4-methylcoumarin

A total of 5.3 g (30 mmol) of 7-Hydroxyl 4-methylcoumarin, 6.4 mL (60 mmol) of 2-bromoethanol, and 8.4 g (60 mmol) of potassium carbonate were mixed in 100 mL of N, N-Dimethylformamide under nitrogen. The reaction mixture was refluxed for 24 h and then cooled to room temperature. Then, 4 mL of 5% HCl was added until it precipitated a white solid. The mixture was extracted and the white crude product solid removed. The crude product solid was recrystallized in ethanol and filtered. The white solid powder of 7- (2-hydroxyethoxy)-4-methylcoumarin was dried in a vacuum (5.28 g, yield of 80%).

### 4.2. Synthesis of 7-(2-Acrylate ethoxy)-4-methylcoumarin

A total of 5 g (20 mmol) of 7-(2-hydroxyethoxy)-4-methylcoumarin and 4 g (40 mmol) of triethylamine were mixed in 50 mL of DCM at 0 °C. Then, 3.6 g (40 mmol) of acryloyl chloride was dissolved in 30 mL of DCM and then dropped into the reaction mixture for two hours under nitrogen. The reaction mixture was stirred at room temperature overnight until the complete consumption of the starting material was observed by TLC (DCM/Hexane, 4/1). After being cooled to room temperature, DCM was removed under reduced pressure. The resulting white solid was dissolved in 10 mL of ethanol. A certain amount of water was added to the solution until it precipitated a white solid. The crude product solid was filtered and recrystallized in ethanol. The pure white powder product was dried in a vacuum and the compound 7-(2-acrylate ethoxy)-4-methylcoumarin (4.7 g, yield of 86%).

### 4.3. Synthesis of Coumarin Containing Copolymer PAC

A total of 9.9 g (130 mmol) of acrylic acid, 3.8 g (13 mmol) of 7-(2-acrylate ethoxy)-4-methylcoumarin, and 0.01 g of AIBN were dissolved in toluene under nitrogen. Next, the reaction mixture was stirred at 80 °C for 24 h. After cooling at room temperature, the mixture was filtered to obtain the solid. The resulting solid was dissolved in water and extracted with chloroform three times. The aqueous solution was collected and the water removed via rotary evaporation to obtain the solid. The solid product PAC was dried in a vacuum (13 g, yield of 95%).

### 4.4. Preparation of the PAC Gel

Due to its highly hydrophilic nature, PAC exhibits good solubility and can easily dissolve in water. Different concentrations of aqueous PAC were prepared and then injected into the mold. These PAC solutions were irradiated with 365 nm UV light (5 mW/cm^2^). Finally, PAC gel was prepared after being irradiated for 1 h.

### 4.5. Characterization of the Sol–Gel Transition Behavior

PAC sol solution with concentrations of 0.1 wt%, 0.5 wt%, 1 wt%, 5 wt%, 10 wt%, 12 wt%, 15 wt%, 18 wt%, and 20 wt% were prepared, respectively, and were injected into the mold. They were successively irradiated with 365 nm and 254 nm (100 mW/cm^2^) ultraviolet light to observe their macroscopic changes. The relationship between the sol–gel transition PAC was studied. At the same time, the rheometer test was used to verify the sol–gel state.

### 4.6. Mechanical Performance Test

Tensile performance test. PAC hydrogel was cut into a rectangular sample (20 mm × 10 mm × 2 mm), which was loaded onto the micro in situ mechanical testing machine for a tensile property test. The tensile rate is 50 mm/min. The same sample should be tested five times to calculate the average value.

Load–unload performance test. The gel was stretched with different degrees of strain and then restored to its original length. The stretching rate is 50 mm/min.

### 4.7. Characterization of the Self-Healing Property

PAC gel was cut out of a crack using a knife. First, the wound was irradiated with 254 nm ultraviolet light for 10 min. Then, the wounds were brought into contact with each other, and the incisions were aligned as much as possible. Finally, the wound was irradiated with 365 nm ultraviolet light (100 mW/cm^2^) for 20 min. Tensile tests were performed to assess the mechanical properties of the healed material. The self-healing process was observed using an inverted fluorescence microscope.

### 4.8. Adherence Test

PAC solution was applied onto one glass slide. One end of the other piece of glass was bonded to the PAC solution. Firstly, the initial adhesion of the sample was tested via tensile testing. Then, the same sample was irradiated with 365 nm (100 mW/cm^2^) for 20 min, which continued to test the adhesion. Finally, the sample was irradiated with 254 nm (100 mW/cm^2^) for 10 min, which tested the adhesion again. Therefore, adhesion strength at each stage was obtained.

## Data Availability

All data and materials are available on request from the corresponding author. The data are not publicly available due to ongoing research using a part of the data.

## Supplementary Materials

The following supporting information can be downloaded at https://www.mdpi.com/article/10.3390/gels10010021/s1: Scheme S1: Synthetic route of PAC; Figures S1–S3: ^1^H-NMR, ^13^C-NMR, and mass spectrum of 7-(2-hydroxyethoxy)-4-methylcoumarin; Figures S4–S6: ^1^H-NMR, ^13^C-NMR, and mass spectrum of 7-(2-acrylate ethoxy)-4-methylcoumarin; Figure S7: ^1^H-NMR spectrum of PAC; Figure S8: Photoresponse recycle of PAC; Figure S9: Emission spectra of PAC solution versus time under UV irradiation; Figure S10: Viscosity of PAC solution with different concentration; Figure S11: Load–unload stress–strain curves of PAC gel with different strains; Figure S12: Tensile stress–strain curves of PAC gel.
