# Supplementary material for: A Sol–Gel Transition and Self-Healing Hydrogel Triggered via Photodimerization of Coumarin
Source: Gels. 2023 Dec 26;10(1):21. doi: 10.3390/gels10010021 (PMC10815305; doi:10.3390/gels10010021)
Supplement: Supplementary file 1 [file gels-10-00021-s001.zip › gels-2761125-supplementary.pdf]

Supporting Information

A Sol-Gel Transition and Self-healing hydrogel triggered via Photodimerization of Coumarin

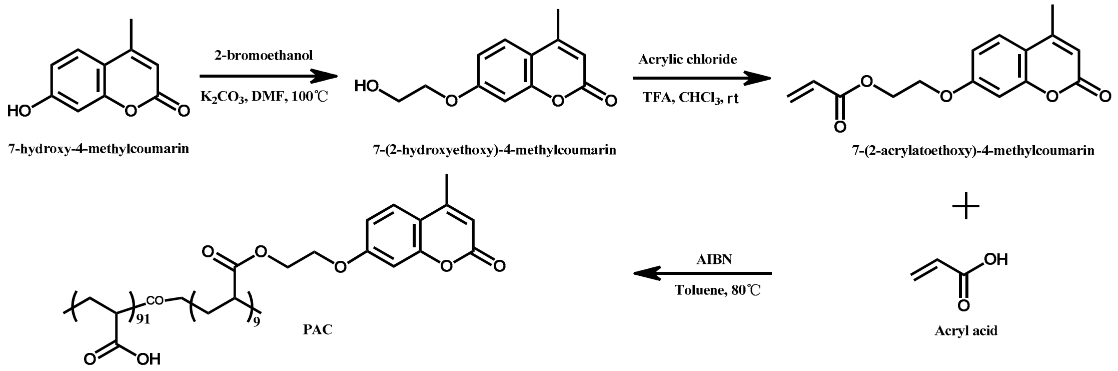

Scheme S1. Synthetic route of PAC.

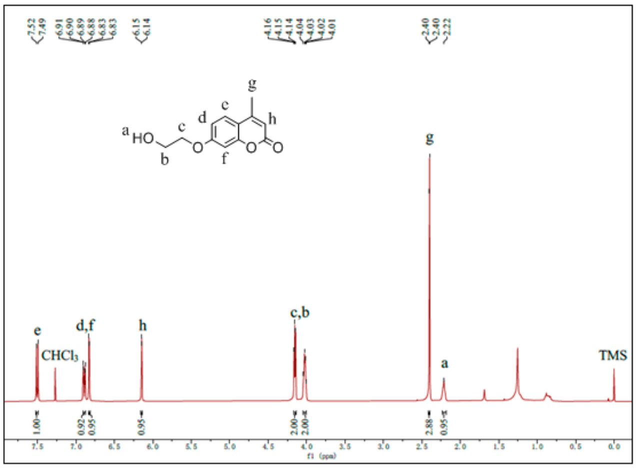

Figure S1. ^{1}H-NMR spectrum of 7-(2-hydroxyethoxy)-4-methylcoumarin in CD_3Cl at 25°C (TMS standard).

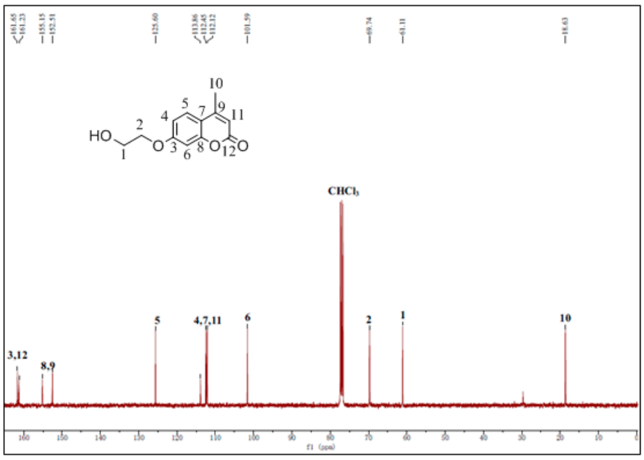

Figure S2. ^{13}C-NMR spectrum of 7-(2-hydroxyethoxy)-4-methylcoumarin in CD_3Cl at 25°C (TMS standard).

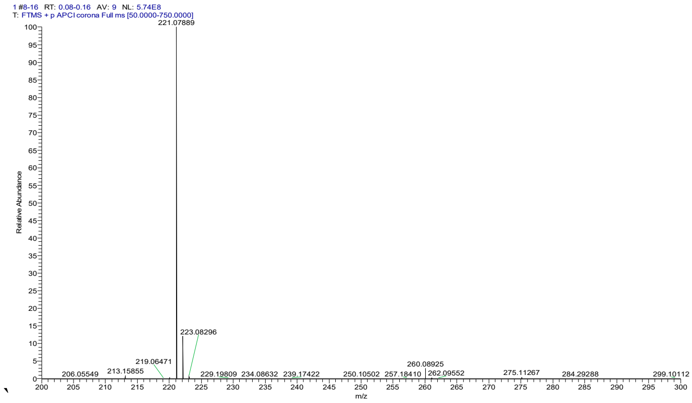

Figure S3. Mass spectrum of 7-(2-hydroxyethoxy)-4-methylcoumarin.

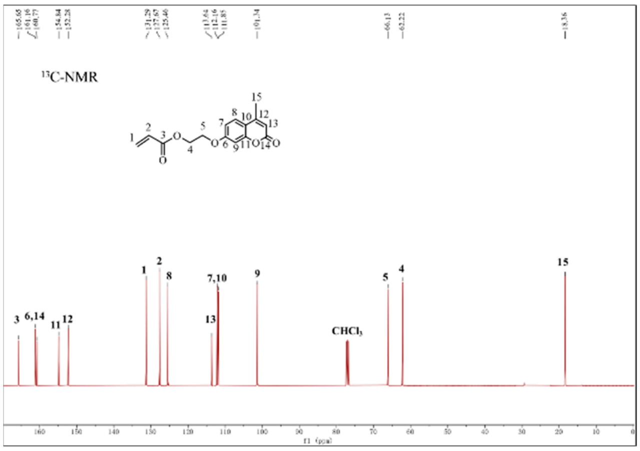

Figure S4. ^{1}H-NMR spectrum of 7-(2-acrylate ethoxy)-4-methylcoumarin in CD_3Cl at 25°C (TMS standard).

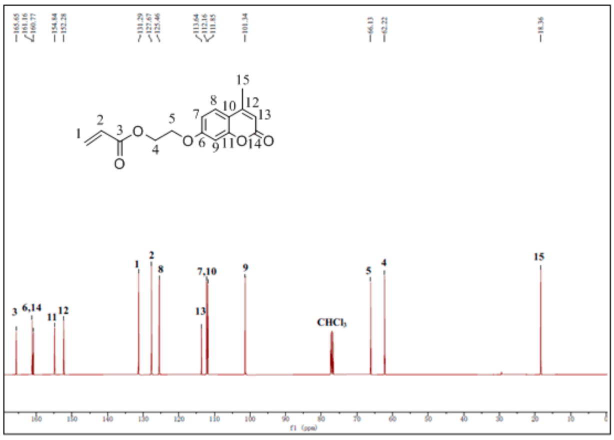

Figure S5.^{13}C-NMR spectrum of 7-(2-acrylate ethoxy)-4-methylcoumarin in CD_3Cl at 25°C (TMS standard).

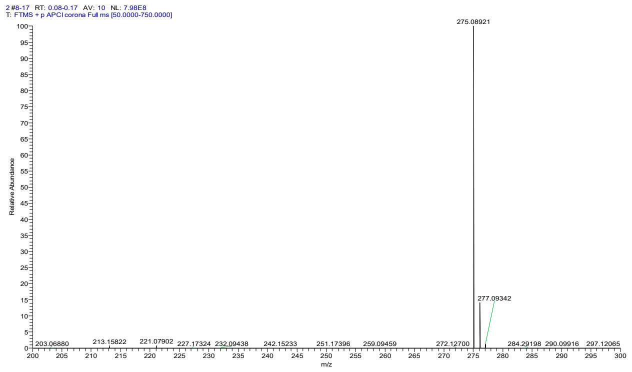

Figure S6. Mass spectrum of 7-(2-acrylate ethoxy)-4-methylcoumarin.

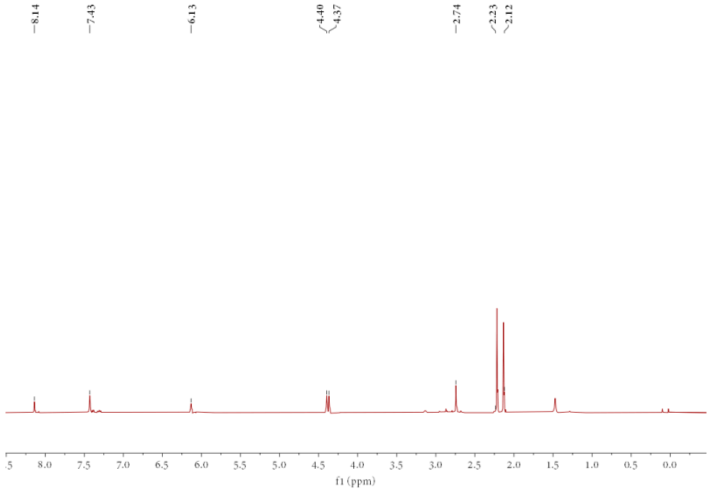

Figure S7. ^{1}H-NMR spectrum of PAC in CD_3Cl at 25°C (TMS standard).

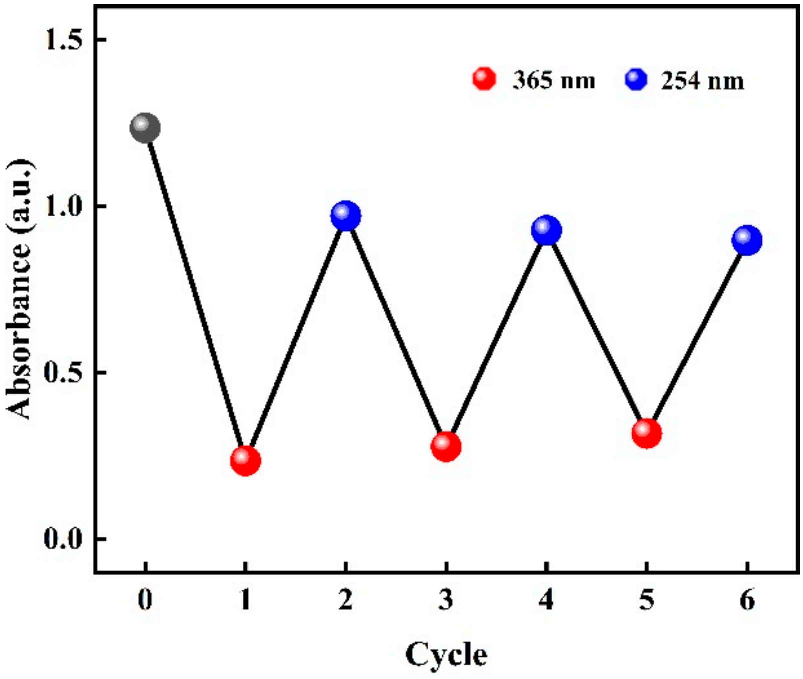

Figure S8. Photoresponse recycle of PAC.

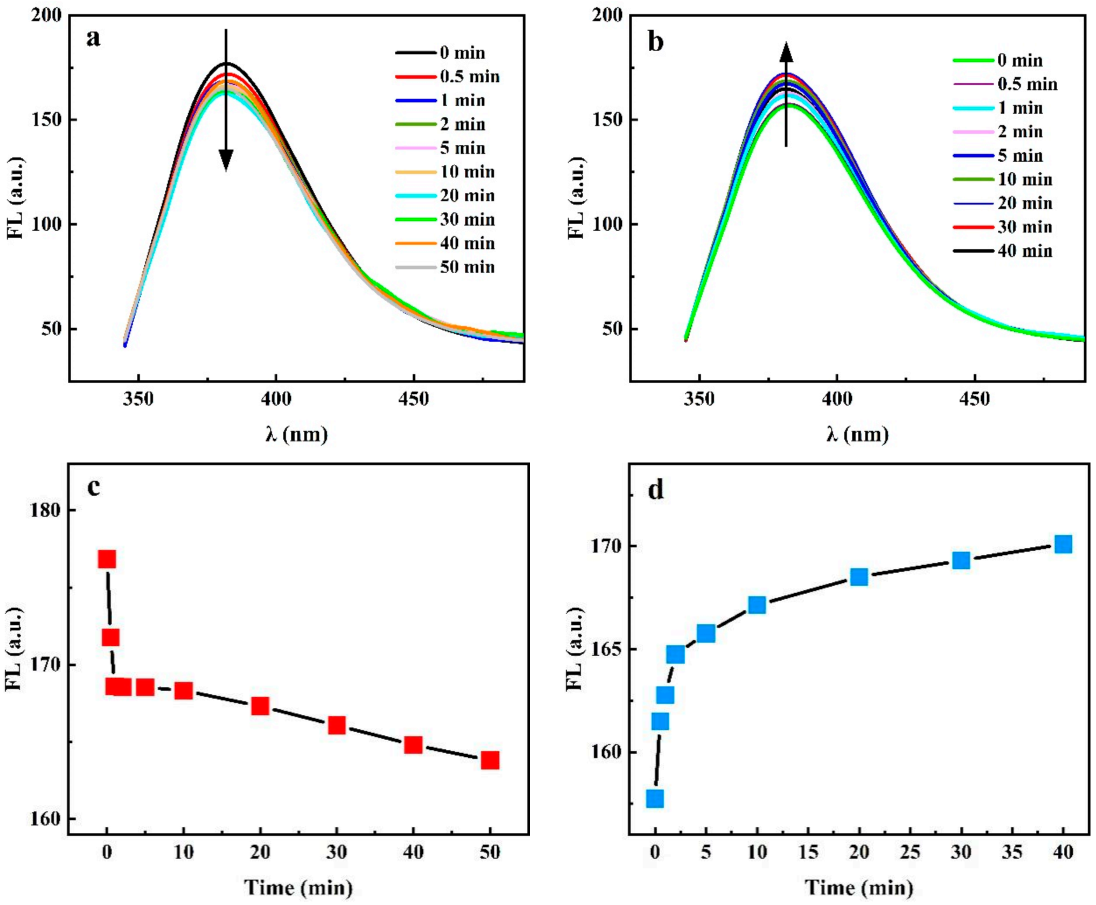

Figure S9. Emission spectra of PAC solution versus time under 365 nm (a) and 254 nm (b) UV irradiation, respectively. Emission intensity at 382 nm with time under 365 nm (c) and 254 nm (d) UV irradiation, respectively. (Concentration: 0.0001 g/ml, λ ex = 300 nm, slit 5 nm)

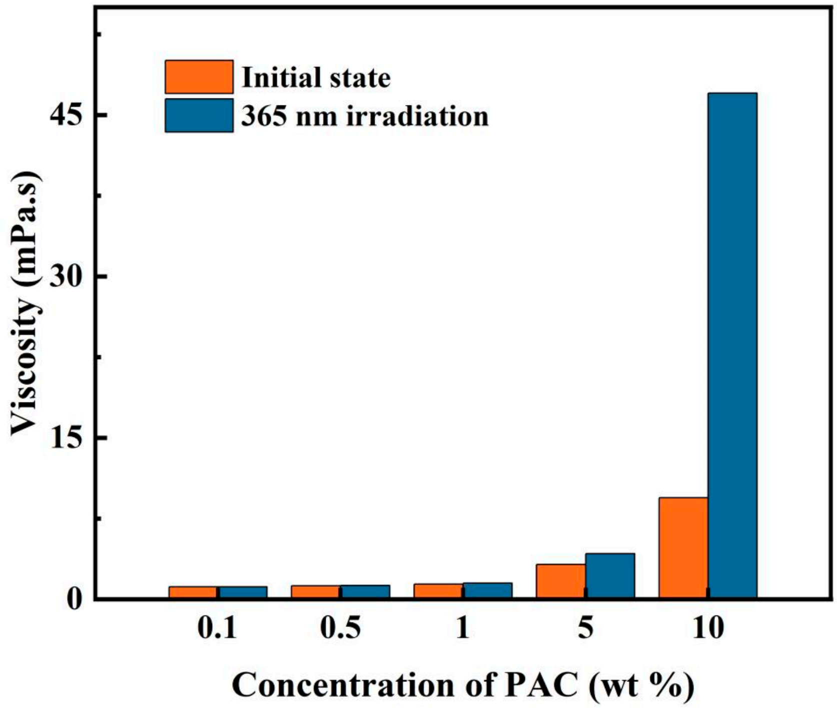

Figure S10. Viscosity of PAC solution with different concentration.

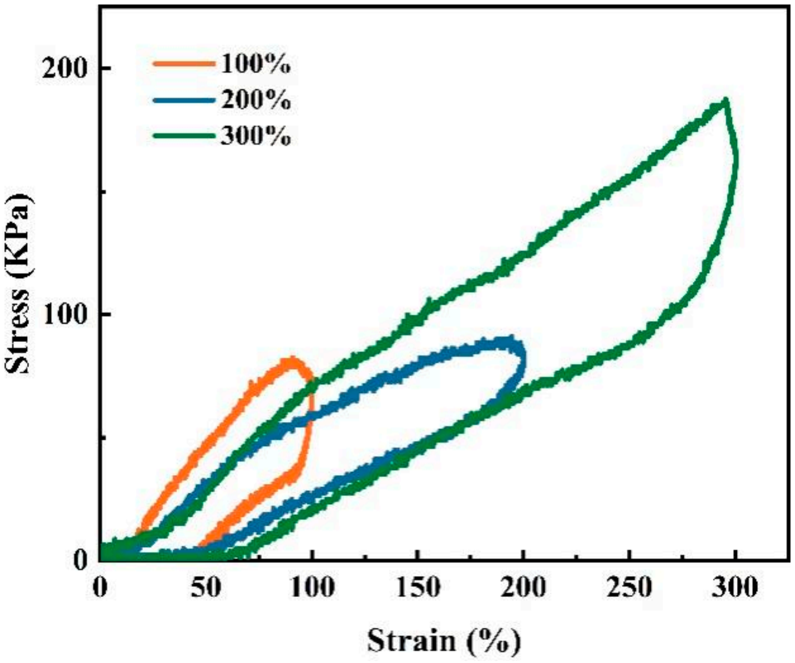

Figure S11. Load-unload stress-strain curve of PAC gel with 100%, 200%, and 300% strain.

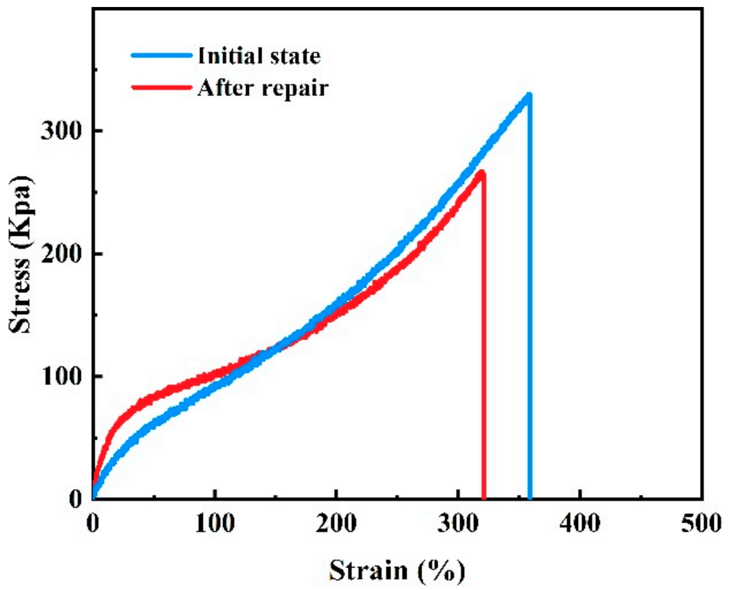

Figure S12. Tensile stress-strain curves of PAC gel (blue line is the initial, and red line is value of repaired gel).
